# Get Back, a person-centred digital programme targeting physical activity for patients undergoing spinal stenosis surgery—a study protocol of a randomized feasibility study

**DOI:** 10.1186/s40814-023-01433-9

**Published:** 2024-01-26

**Authors:** Emelie Karlsson, Rikard Hanafi, Helena Brisby, Andreas Fors, Mike Kemani, Håkan Hedman, Jo Nijs, Mari Lundberg

**Affiliations:** 1 Back in Motion Research Group, Department of Health Promoting Science, Sophiahemmet University, Box 5605, 114 86 Stockholm, SE Sweden; 2 Pain in Motion Research Group (PAIN), Department of Physiotherapy, Human Physiology and Anatomy, Faculty of Physical Education & Physiotherapy, Vrije Universiteit Brussel, 1050 Brussels, Belgium; 3 Karolinska University Hospital, Theme Women’s Health and Allied Health Professionals, Medical Unit Medical Psychology, Solna, Sweden; 4 Department of Orthopaedics, Institute of Clinical Sciences at Sahlgrenska Academy, University of Gothenburg, Gothenburg, Sweden; 5 Department of Orthopaedics, Sahlgrenska University Hospital, Gothenburg, Sweden; 6 Institute of Health and Care Sciences, Sahlgrenska Academy, University of Gothenburg, Gothenburg, Sweden; 7 University of Gothenburg Centre for Person-Centred Care (GPCC), Sahlgrenska Academy, University of Gothenburg, Gothenburg, Sweden; 8 Region Västra Götaland, Research, Education, Development and Innovation, Primary Health Care, Gothenburg, Sweden; 9 Department of Clinical Neuroscience, Karolinska Institutet, Stockholm, Sweden; 10 Chronic Pain Rehabilitation, Department of Physical Medicine and Physiotherapy, University Hospital Brussels, 1050 Brussels, Belgium; 11 Department of Health and Rehabilitation, Unit of Physiotherapy, Institute of Neuroscience and Physiology, Sahlgrenska Academy, University of Gothenburg, Gothenburg, Sweden

**Keywords:** Spine disease, eHealth, Physical activity, Physiotherapy, Feasibility

## Abstract

**Background:**

Spinal stenosis is the most common reason for elective spine surgery, and the cardinal symptom is leg pain and discomfort when walking. Patients with spinal stenosis have a decreased level of physical activity and thereby an increased risk of poor health. Get Back is a person-centred digital programme that strives to support patients being physically active after surgery. The aim is to explore if Get Back, in its present format (referred to as Get Back_feasibility_), is feasible and contributes to detectable change in variables related to intervention content.

**Methods:**

Thirty patients planned for decompression surgery due to central lumbar spinal stenosis who present with low physical activity, pain catastrophizing or fear of movement, will be included in a randomized feasibility study. All patients will be randomly allocated to either Get Back_feasibility_ or usual physical therapy. Get Back_feasibility_ aims to increase the patient’s physical activity level by combining a person-centred and cognitive behavioural approach. It comprises 10 video and telephone sessions led by a physical therapist over 12 weeks (pre/postoperatively). Outcomes are treatment fidelity (treatment dose, adherence, and content), process feasibility (recruitment, intervention use, and acceptability of measurements and intervention), and variables related to the intervention content (steps per day, physical activity level, pain catastrophizing, fear of movement, and general self-efficacy). Treatment fidelity and feasibility data will be assessed during the full study period (12 weeks). Physical activity, physical capacity, and patient-reported outcomes will be assessed digitally at baseline (2 weeks preoperatively) and 11–12 weeks postoperatively. Variables related to the intervention content will be monitored weekly through a digital application. Feasibility data will be analysed descriptively and inferentially using a nonparametric approach, data from repeated measures will be displayed graphically and data from telephone interviews will be analysed using content analysis with a descriptive manifest approach.

**Discussion:**

The results will provide information on whether Get Back in its present format is feasible and can be evaluated for effectiveness in a larger randomized controlled trial, for patients with a low physical activity level and a high fear of movement who are undergoing decompression surgery.

**Trial registration:**

Registered at ClinicalTrails.gov 04/08/2023, registration no. NCT05806593.

**Supplementary Information:**

The online version contains supplementary material available at 10.1186/s40814-023-01433-9.

## Background

Lumbar spinal stenosis (LSS) is the most common indication for surgical treatment in the lumbar spine [1]. LSS often causes neurogenic claudiocation which in turn limits walking ability. The surgical preference for LSS is decompression [2] aiming at relieving pain, and increasing walking ability and health-related quality of life. Studies suggest that patients with LSS are less likely to meet recommendations for healthy physical activity before and after surgery compared to population norms [3, 4]. Physical inactivity before surgery for LSS is associated with less improvement in postoperative disability and pain [5]. Since people who are physically inactive have an increased risk for noncommunicable diseases such as cardiovascular disease, cancers, and diabetes [6], physical activity should be included in pre- and rehabilitation programmes. It is known that people with the lowest levels of physical activity have the largest health-related benefits of increasing their activity [7].

A recent meta-analysis assessing the effect of prehabilitation prior to lumbar spine surgery (LSS included) concluded that there was very low to low certainty for evidence of no additional effect compared to usual care on postoperative physical functioning, pain, and complications [8]. Nevertheless, studies in the meta-analysis included study populations with both high- and low-risk patients, a variety of interventions and the sample sizes were small. One way to reinforce certainty is to target high-risk subgroups, such as people who are deconditioned with low physical activity, in future studies [8]. In addition, a need has been pointed out for studies investigating the combination of pre- and rehabilitation, as the optimal rehabilitation period is still unclear [9, 10].

Previous pre- and rehabilitation programmes for LSS have primarily focused on reducing disability rather than promoting health [11, 12]. A global call suggested health as an overarching strategic approach for people with low back pain [13]. Physical activity is identified as a significant indicator for health and can be used as a valid outcome. To date, outcomes have primarily been evaluated with patient-reported outcome measurements (PROMs) within the research field [14], and it has been questioned how well they capture postoperative physical activity and related health [15].

For patients with lumbar spinal stenosis, the cardinal functional problem is walking, and poorer walking capacity is associated with lower daily step counts [16]. Since steps per day is associated with a progressively lower risk of all-cause mortality up to 6000–8000 steps per day in people over 60 years of age [17], walking is recommended directly after surgery. Increased walking time and steps per day directly after surgery are associated with reduced pain and opioid use, as well as improved functioning 6 and 12 months after lumbar spine surgery due to a lumbar degenerative condition including LSS [18, 19]. In the current study, steps per day will be the main focus.

Common reasons, as described by patients, for not engaging in physical activity post-surgery are sedentary habits, persistent pain, and fear of reinjury of the spine [20]. There is conflicting evidence regarding the association between preoperative fear of movement/pain catastrophizing and postoperative pain and disability [21–23], and little is known about how fear of movement interacts with physical health outcomes such as physical activity. In a prior study, we found that preoperative fear of movement was a significant predictor of sedentary behaviour at 6 and 12 months after lumbar fusion surgery. A few studies have indicated that pain-related fear of movement is associated with fewer steps per day in patients with degenerative lumbar disease, including LSS [16, 24].

Moreover, it is suggested that “one size does not fit all” [25], and a person-centred approach is recommended [13]. Person-centred care (PCC) has been shown to increase patients’ levels of self-efficacy in other pain conditions [26]. Therefore, PCC will be a key component of the Get Back programme. Get Back comprises the further development of a previously evaluated person-centred prehabilitation programme including a cognitive behavioural approach that promoted physical activity for patients with chronic low back pain undergoing lumbar fusion surgery [27]. In our prior prehabilitation study, physical activity did not improve over time, despite significantly improved self-reported functioning and objectively measured physical capacity. These findings have an impact on the current understanding of the long-term effects of prehabilitation and of future research, which should focus on programmes promoting physical activity both before and after lumbar spine surgery to decrease the risk of long-term adverse health outcomes (Kemani et al. Long-term follow-up of a Person-Centred Prehabilitation Program Based on Cognitive-Behavioural Physical Therapy for Patients Scheduled for Lumbar Fusion Surgery. *Submitted*). Get Back will be delivered in an eHealth format. It has previously been revealed that patients decline participation in face-to-face interventions due to geographical barriers [27]. The use of eHealth can support their availability for rehabilitation [28].

Due to the argumentation above, Get Back is designed in the format of a person-centred digital programme that aims to support high-risk patients in being physically active after decompression surgery for spinal stenosis. Prior to performing a full-scale randomized controlled trial, we will conduct a feasibility trial to assure and refine the evaluation design and the intervention itself, as recommended by The Medical Research Council [29]. The current version of the intervention, tested in the feasibility study, will be referred to as Get Back_feasibility_. Here we present the protocol of a study with the objective of evaluating whether Get Back_feasibility_ in combination with decompression surgery can provide a detectable change in variables related to the intervention content and to evaluate the treatment fidelity as well as feasibility in terms of the trial procedure, intervention use, and acceptability in patients identified with a low physical activity level and pain catastrophizing or fear of movement.

## Methods

### Research questions

All research questions below are formulated in the PICO format and concern patients with a low physical activity level and high pain-related catastrophizing and/or fear of movement receiving decompression surgery for lumbar spinal stenosis.

#### Research questions pertaining to outcomes relating to the Get Back_feasibility_ content

1. Do the assessments preoperatively and at 12-week follow-up of steps per day, physical activity and pain catastrophizing, fear of movement and general self-efficacy provide tentative information as to the efficacy of the Get Back_feasibility_ intervention?
2. Do the weekly assessments of single-item questions aiming to measure steps per day, physical activity and aspects of pain catastrophizing, fear of movement and self-efficacy provide additional information regarding the efficacy of the Get Back_feasibility_ intervention, trajectories of change and interrelations between variables?

#### Research questions in relation to treatment fidelity of the Get Back_feasibility_ intervention

(3) Is the treatment dose and content of Get Back_feasibility_ delivered as intended?
(4) Does the physical therapist delivering the Get Back_feasibility_ intervention adhere to a person-centred approach?

#### Research questions in relation to process and resource feasibility

(5) What percentage of patients planned for decompression surgery for lumbar spinal stenosis meeting inclusion criteria are eligible after the screening procedure?
(6) What are the reasons for declining participation in the study or dropping out?
(7) Is the screening questionnaire measuring physical activity level able to detect patients with a low level of physical activity compared to accelerometer data at baseline?
(8) How many of the planned sessions of the Get Back_feasibility_ intervention do patients of the intervention group attend?
(9) Did the study participants and physical therapists (PT) in the study find the digital format, Get Back_feasibility_ intervention, and outcome measures relevant and usable?
(10) Is the Get Back_feasibility_ treatment safe (type and frequency of adverse events)?
(11) What is the response rate of the used PROMs and to what extent are physical tests completed? If they are not completed, what are the reasons?

### Trial design

A randomized (1:1 allocation ratio) feasibility study design will be used. The protocol will be reported in accordance with the Standard Protocol Items: Recommendations for Interventional Trials (SPIRIT) [30] (for a checklist please see Additional file 1) and with further guidance from the Consolidated Standards of Reporting Trials (CONSORT) extension for randomized pilot and feasibility trials [31].

### Patient involvement

Patients and members of the public will be involved as research partners throughout the research process in accordance with Patient and Public Partnership guidelines [32]. During the preparation and feasibility phase one patient representative joined the steering group, was a coapplicant on the funding application, provided feedback on parts of the ethical application (summaries, patient information, and informed consent), and tested the usability of the accelerometer and related written instructions. The patient representative will be regularly updated on the status of the research process and informed via email communication once the feasibility trial has been published. After the feasibility study, additional patient representatives will be recruited to work together with the researchers during the process of finalizing the design of the full trial.

### Participants and study settings

Participants will be recruited from two private spine clinics with referrals from the regions, in Sweden: Capio Spine Center Göteborg and Ryggkirurgiskt centrum (RKC), Sophiahemmet, Stockholm. Inclusion criteria: (i) patients planned for decompression surgery (without concomitant fusion) due to lumbar central spinal stenosis; (ii) patients > 18 years of age. Exclusion criteria: (i) patients with malignancy, severe neurological -or rheumatological disease, idiopathic scoliosis, or isthmic spondylolisthesis; (ii) persons unable to understand written information and communicate in Swedish; and (iii) patients with untreated/unstable heart disease that prohibits physical capacity tests. For patients meeting the inclusion criteria, the following screening criteria should also be fulfilled: (i) low level of physical activity (i.e. persons who do not meet WHO’s physical activity recommendations of a weekly minimum of 150 min of physical activity of moderate intensity), and (ii) higher levels of fear of movement or pain catastrophizing, equivalent with scores ≥ 37 on the Tampa Scale of Kinesiophobia (TSK) [33] and ≥ 30 on the Pain Catastrophizing Scale (PCS) [34].

### Recruitment

**Fig. 1:**
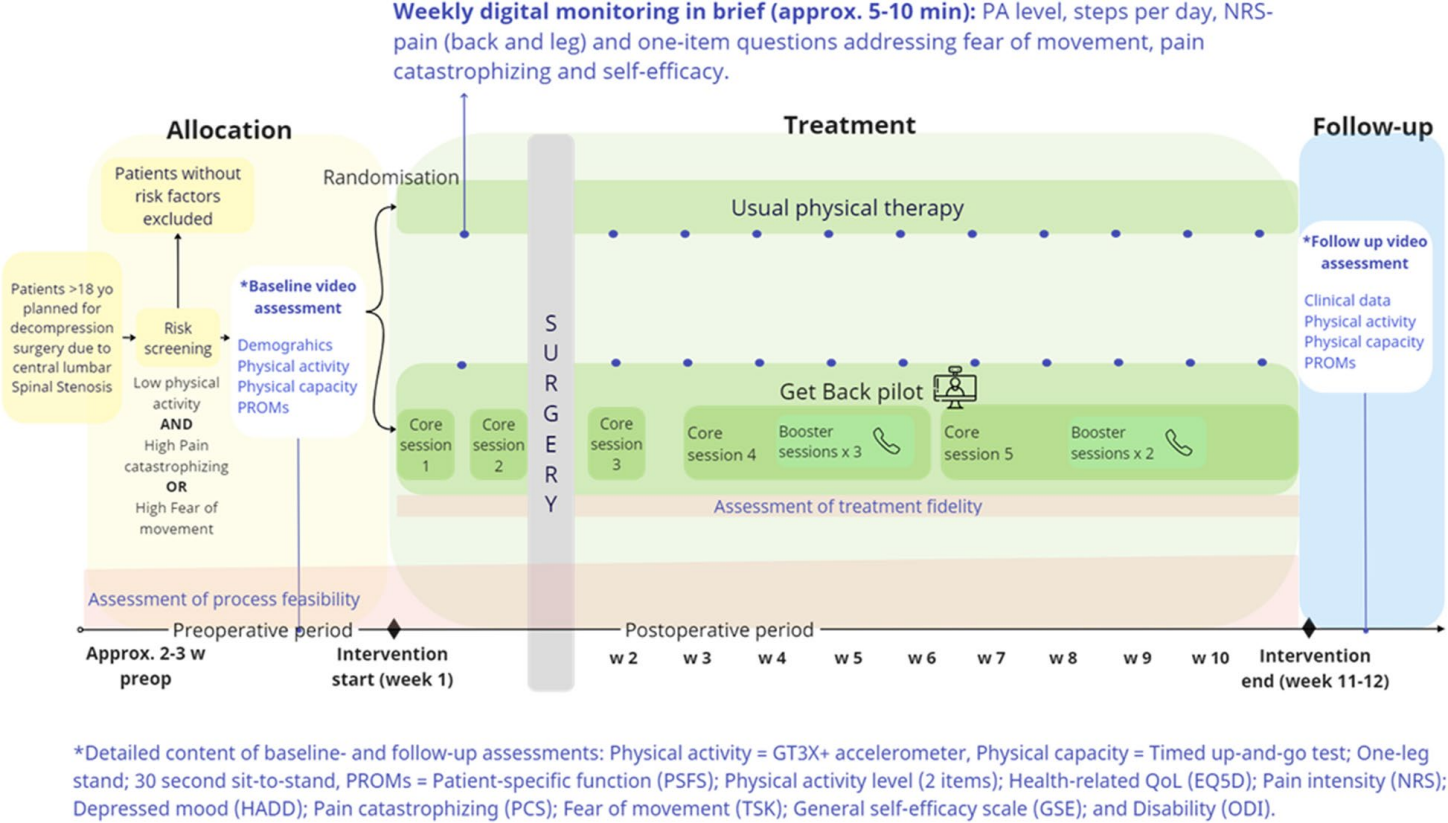
Study flow including allocation, treatment, and follow-up phases

All patients will have a consultation with a spine surgery specialist who based on anamnesis and clinical and radiological findings will determine the diagnosis and give recommendations regarding the treatment regime(s). Patients planned for surgery and eligible for the study will be identified by an independent local recruiter who will contact the patients by phone, provide oral study information and screen for risk factors. The independent recruiter will also ascertain whether the participant has the required technology (smartphone, computer, or tablet with a camera, as well as an internet or mobile network connection). If the screening criteria are fulfilled, the patient will be asked for participation and oral informed consent. A digital baseline assessment will be scheduled with an independent observer (physical therapist) approximately 2 weeks before surgery. Before the baseline assessment, written informed consent will be obtained digitally. An overview of the study flow is seen in Fig. 1.

### Randomization

A statistician will generate one computerized list per recruitment site with the random allocation sequence. A person not involved in either recruitment or data collection will administer the lists into concealed envelopes. The concealed and numbered envelopes will be consecutively opened by a study coordinator after each patient’s baseline assessment and allocate the patients to either Get Back_feasibility_ (*n* = 15) or usual physical therapy (*n* = 15). The independent observer will be blinded to the group allocation. Participants allocated to the intervention will not participate in physical therapy at the operating clinic from postoperative discharge and as long as the Get Back intervention is conducted (approximately 10–11 weeks).

### Theoretical framework of the intervention

Get Back combines a person-centred philosophical standpoint [35, 36] with a behavioural medicine perspective [37] on pre- and rehabilitation. The overall aim is to improve physical activity as a core indicator for health. Physical activity should be promoted for patients with chronic pain as well as for patients with disorders affecting mobility in combination with pain, such as spinal stenosis patients. First, physical activity decreases the risk for noncommunicable diseases and thereby decreases the risk for premature death and years lived with disability [38]. Second, physical activity has a hypoalgesic effect [39].

PCC, as defined by the framework developed by the University of Gothenburg Centre for Person-Centred Care (GPCC) [35], is an approach based on ethical principles that aims to involve patients as active partners in their care and treatment and establish a partnership reflecting the expertise of both the patient and health professionals [35, 36]. The core component of PCC is the cocreation of care through partnership between patients, their families and informal carers, and health professionals. Since 2010 the GPCC framework has been operationalized and evaluated in several controlled trials [40] and implemented in clinical practice based on three routines. These routines are based on listening to the patient’s narrative to identify personal needs and resources, together with medical status, to initiate a partnership; working the partnership to achieve commonly agreed goals; and safeguarding the partnership by documenting a jointly agreed health plan. Within other research fields, e.g. in patients recovering from acute coronary syndrome and patients with common mental disorders, it has been indicated that person-centred care increases self-efficacy regarding symptom control respectively in general [26, 41].

The treatment content is developed based on learning theory and, specifically, the fear-avoidance model of pain [33, 42]. In short, the model explains how, depending on the threat interpretation of pain, a person can either prioritize avoiding activities that could further elicit the pain or prioritize valued life goals and behaviours leading to recovery. Exposure aims to tackle avoidance behaviour to improve functioning through increased approach behaviour, not fear-reduction per se [43]. Pain communication (pain neuroscience education, PNE) aims to reconceptualize and undermine the threat value of an individual’s pain experience [44]. In Get Back_feasibility_, pain communication is conceptualized as a prerequisite to an active exposure approach to physical activity and not an independent intervention.

### Intervention content and procedure

**Fig. 2:**
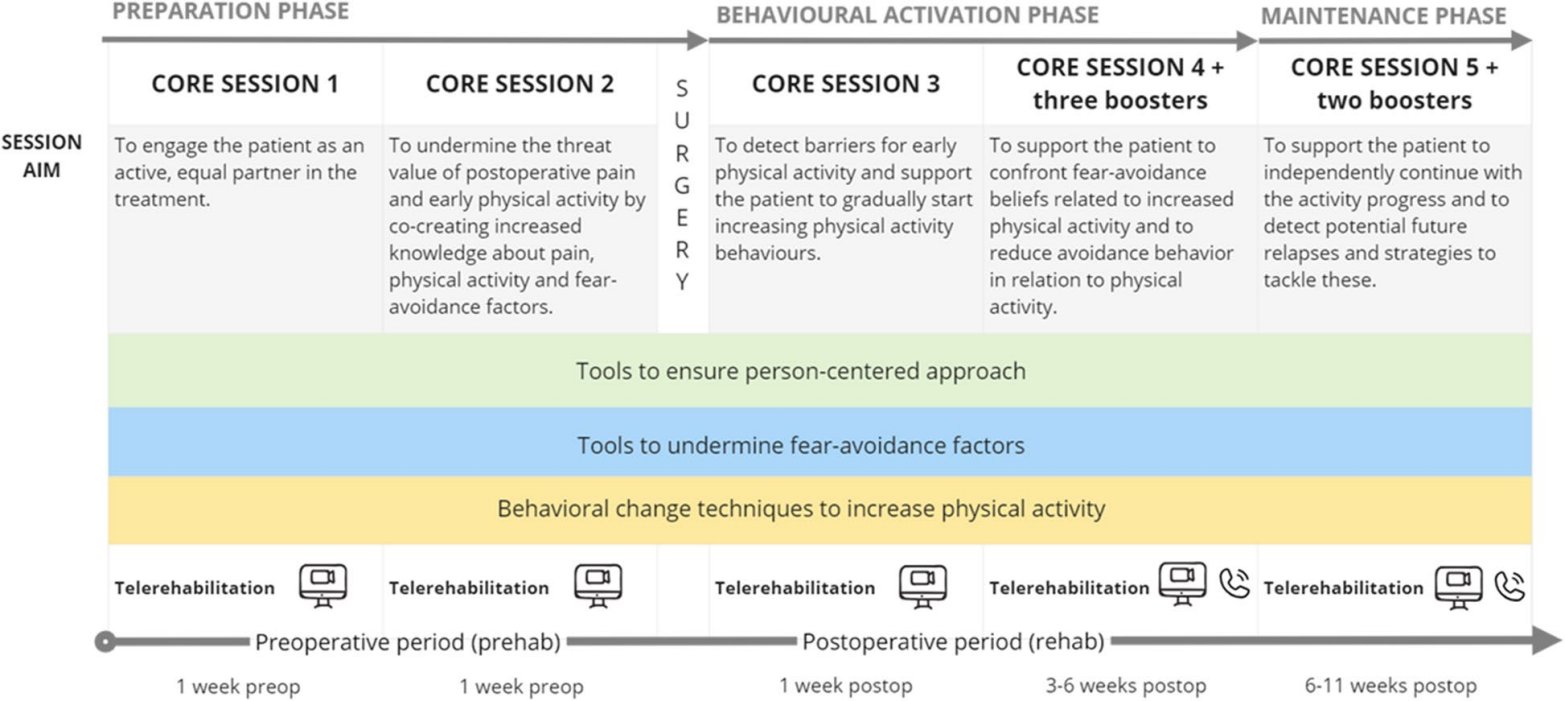
An overall schematic illustration of the intervention (Get Back_feasibility_)

Get Back_feasibility_ will be delivered by a physical therapist (Study PT) via video and telephone, enabling patients to access the intervention from their homes. Video communication will be conducted on the Doctrin platform (provided by Doctrin AB), which is an approved medical device, and all data will be processed according to the Swedish Patient Data Act and the EU Data Protection Regulation (GDPR). Get Back_feasibility_ includes five treatment sessions with predefined aims and content delivered through videocall (core sessions) and five shorter reinforcing telephone sessions (booster sessions). Each core session will last for approximately 1 h, and each booster session will last < 30 min. Out-of-session tasks [45] will be formulated at every session and followed up at the next. The intervention will span over 12 weeks (1 week preoperatively until 11 weeks postoperatively). The Study PT will have experience and education in PCC and pain. All parts of the intervention are based on a person-centred approach and are theoretically based on the modified version of the fear-avoidance model [27, 42]. Should unexpected findings of a medical nature be observed during any of the sessions with the study physical therapist, these will be discussed with medical experts in the research group. The sessions and included Behavioural Change Techniques (BCTs) according to the BCT taxonomy [46] are described below and illustrated in Fig. 2.

#### Preparation phase—initiating and safeguarding partnership

*Core session 1: Person-centred analysis and treatment start-up (1 week preoperatively)*

The focus of the first core session is to engage the patient as an active, equal partner in the treatment and to formulate a shared health plan according to PCC [35]. The health plan will be based on the results from baseline measurements and on actively listening to the patient’s narrative. Using person-centred communication (e.g. active listening, open-ended questions) [47] and the modified fear-avoidance model as tools, the focus will be on identifying the persons’ unique resources, needs and challenges for engaging in physical activity. The shared health plan will further include reasonable and reachable goals, both personally valued long-term physical activity outcomes and more short-term everyday activity goals, as well as strategies and specific exercises needed to reach each goal.

Examples of out-of-session tasks:

- Self-monitoring [45] steps per day as a behaviour reinforcement tool [48, 49].
- Reading information material in preparation for session 2 [50].

*BCTs include* goal setting (behaviour and outcome) (1.1, 1.3), action planning (1.4), feedback on behaviour (2.2), and self-monitoring of behaviour (2.3).

*Core session 2: Person-centred preoperative communication (1 week preoperatively)*

The focus of core session 2 is to undermine the threat value of postoperative pain and early physical activity by cocreating increased knowledge about pain, physical activity, and fear-avoidance factors [51]. The structure of the session will be based on recommendations by Goudman et al. [52] and tailored to fit the patient’s own preferences and questions arising from session 1, in line with PCC. The evidence-base for Get Back will be discussed: Healthy physical activity behaviour post-surgery can improve recovery and improve health and quality of life. Moreover, that postoperative pain is normal, expected and not an accurate reflection of the result of surgery. The Study PT will ensure the patient that the recommended physical activity will be in line with potential individual postoperative recommendations. The health plan will be revisited and documented. The PT will follow up on the activity-tracker, give reinforcing feedback on the behaviour and engage in problem solving if needed.

Examples of out-of-session tasks:

- Self-monitoring [45] steps per day as a behaviour reinforcement tool [48, 49].

*BCTs include* action planning (1.4), feedback on behaviour (2.2), self-monitoring of behaviour (2.3), information about health consequences (5.1), and instructions on how to perform a behaviour (4.1).

#### Behavioural activation phase—working and safeguarding partnership

*Core session 3: Person-centred postoperative analysis and activity initiation (1 week postoperatively)*

The focus of this core session is to detect barriers for early postoperative physical activity and help the patient to gradually start increasing physical activity behaviours after surgery. The preoperative pain- and physical activity communication from session 2 will be followed up based on the patients’ current thoughts and coping strategies related to postoperative pain and movement [52]. Behaviour analysis, using the fear-avoidance model as an exemplifying tool, will be used to cocreate an understanding of the patient’s postoperative physical activity behaviour and its consequences. Then, in partnership, the patient and PT will plan for a graded increase in physical activity using self-monitoring and out-of-session tasks [45]. As walking is the recommended activity directly post-surgery, the focus will be on walking and increasing the number of steps per day. The patient’s own activity goals will be incorporated in the planning. All recommendations on early postoperative physical activity will be in line with individual postoperative regime made by the surgeon. The health plan, including personal goals, will be revisited and revised if needed.

Examples of out-of-session tasks:

- Behavioural experiment—the patient will be encouraged to test a planned increase in physical activity behaviour, along with preformulated hypotheses about the consequences and collection of data for the results to be analysed in session 4.
- Self-monitoring [45] steps per day as a behaviour reinforcement tool [48, 49].

*BCTs include* action planning (1,4), review of behaviour goals (1.5), behavioural experiments (4.4), graded tasks (8.7), exposure (7.7), behavioural rehearsal/practice (8.1), and self-monitoring of behaviour (2.3).

*Core session 4 + Booster sessions 1–3: Reinforcing physical activity (3–6 weeks postoperatively)*

The focus of this core session is to help the patient to further confront fear-avoidance beliefs related to increased physical activity behaviour and to undermine avoidance behaviour in relation to physical activity**.** The patient and PT will revisit the patient’s experience of being physically active in terms of thoughts, feelings, and short- and long-term consequences of a given behaviour. The PT will provide reinforcing feedback on self-monitoring physical activity behaviour and progress on daily activity goals. Behavioural analysis will be performed using the fear-avoidance model as an exemplifying tool to increase a shared understanding of the patients’ physical activity behaviour and related thoughts and consequences. The short- and long-term consequences of approach or avoidance behaviour will be discussed in relation to the persons’ long-term values in relation to physical activity. In partnership, the patient and PT will then plan for the patient to systematically confront fears and/or other potential barriers with the aim of decreasing avoidance and increasing approach behaviour related to physical activity (exposure) [43]. The Study PT will offer to be on the phone with the patient during the exposure as a facilitating factor. The health plan will be revisited and revised if needed. Depending on whether the patient reaches the set goals or experiences setbacks, new goal setting will be adapted for each patient’s situation.

Examples of out-of-session tasks:

- Exposure for avoidance behaviour related to physical activity.
- Self-monitoring [45] steps per day as a behaviour reinforcement tool [48, 49].

*BCTs include* a review of behaviour goals (1.5), self-monitoring of behaviour (2.3), behavioural experiments (4.4), exposure (7.7), behavioural rehearsal/practice (8.1), and graded tasks (8.7).

#### Maintenance phase—maintaining and safeguarding partnership

*Core session 5 + booster sessions 4–5: Person-centred activity maintenance plan (6–11 weeks postoperatively)*

The focus of this core session is to help the patient to independently continue with the activity progress as well as to detect potential future relapses and discuss strategies on how to tackle such setbacks*.* The intervention content will be repeated, and based on the patients’ personal preferences, thoughts and needs. Behavioural- and outcome goals will be evaluated, and the patient will receive reinforcing feedback on progressions in physical activity, everyday activity goals and self-monitoring. The patient will be asked to formulate beneficial strategies learned during the intervention and be encouraged to continue working with these strategies in the long term. Potential future barriers for engaging in physical activity will be lifted, and the patient will be asked to solve how to tackle these barriers based on techniques learned from the intervention and receive feedback. The shared health plan will be updated for long-term recovery, and long-term physical activity goals will be set to enable the patient to sustain long-term healthy physical activity.

*BCTs include* behavioural experiments (4.4), a review of behaviour goals (1.5), problem solving/coping planning (1.2), behavioural rehearsal/practice (8.1), goal setting (1.1, 1.3), habit formation (8.3), habit reversal (8.4), and generalization of a target behaviour (8.6).

### Usual physical therapy

The control group will follow the usual physical therapy, meaning physical therapy as is provided at each recruiting site when patients undergoing surgery due to spinal stenosis. As this may differ substantially between clinical sites nationally, data on the frequency and content of physical therapy sessions during the study period will be collected as a control variable at the weekly assessments from the control group. During hospitalization participants in both groups will receive the usual care regarding physical therapy conducted on-site. Such care can differ between sites, but commonly involves early postoperative mobilization such as transferring from bed to sitting, chair rising, and walking the same or the day after surgery.

### Procedure

**Fig. 3:**
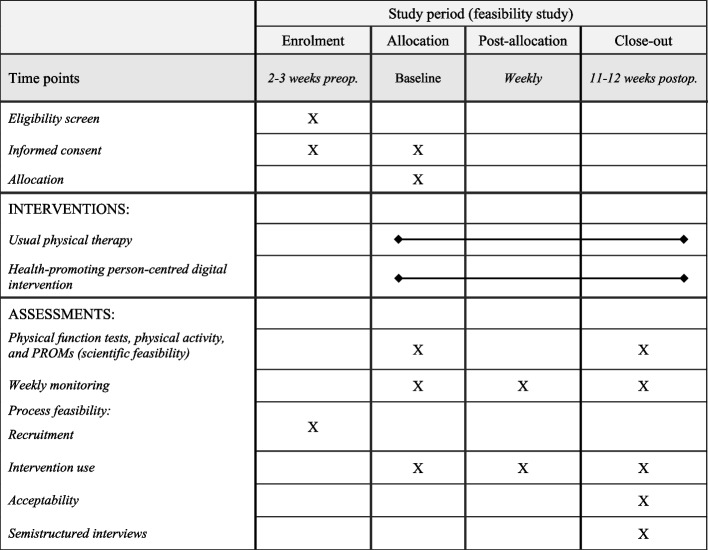
SPIRIT schedule of enrolment, interventions, and assessments

At baseline (approximately 2 weeks preoperatively) and follow-up (11–12 weeks postoperatively), physical activity, physical capacity and PROMs will be assessed. For physical activity, once the patient has agreed to participate in the study, the study coordinator will mail an accelerometer (ActiGraph GT3X + ; ActiGraph, Pensacola, FL, USA) and written user information to the participant for a 7-day assessment. Thereafter, an independent observer will collect demographic/clinical data and conduct physical capacity tests via video. In case of adverse events during video assessments, the independent observer has been instructed to contact medical care immediately if the patient becomes acutely ill or guide the patient to seek medical care if more of a subacute nature. The patient will fill out the PROMs through a secure digital platform (the application BASS, Karolinska Institutet, Stockholm, Sweden). During the intervention, the participants will also fill out weekly repeated measurements in BASS. These weekly measures will include twelve one-item questions addressing physical activity levels, fear of movement, catastrophizing, self-efficacy, and steps per day. Reminders will be sent out automatically via text message if the participant has forgotten to fill in a questionnaire in BASS. At the end of the study, a telephone-based semistructured interview, with all intervention participants, regarding feasibility aspects and participation will be conducted by the study coordinator. Feasibility data will be collected continuously during and at the end of the study period (see Fig. 3).

### Baseline variables and outcome measures

Baseline variables such as age, gender, comorbidity, weight/height, smoking status, educational level, sick-leave status, back and leg pain duration, and previous spine surgery will be collected from the patient. Preoperative cognitive function will be assessed with the Cognitive Performance Scale (CPS) [53]. Pre- and postoperative clinical data such as the type of surgery, analgesic use, complications, length of hospital stay, discharge destination, reoperation, and readmission to the hospital will be collected from the medical records.

#### Physical activity level

- Objectively measured by a digital triaxial accelerometer (ActiGraph) via the variables, steps per day, time spent in light physical activity, moderate to vigorous physical activity (MVPA), and time spent sedentary [54].
- Self-reported physical activity with a two-item questionnaire from the National Board of Health and Welfare in Sweden [55].

#### Physical capacity (digitally)

- Postural balance with the One Leg Test [56, 57]
- Physical function with the Timed Up-and-Go test (TUG) [58]
- Functional leg strength with the 30 s Sit-to-Stand test (30 s STS) [59]

#### Patient-reported outcome measures (digitally)

- The person’s own goals regarding function measured with the Patient-Specific Function Scale (PSFS) [60]
- Health-related quality of life measured with the EQ-5D 3L
- General self-efficacy with the General Self-Efficacy Scale (GSE) [61]
- Pain intensity level in the legs and back reported with the numeric rating scale (NRS)
- Subjective rating of kinesiophobia with the Swedish version of the Tampa Scale of Kinesiophobia (TSK-SV) [62]
- Catastrophizing thoughts measured with the Pain Catastrophizing Scale (PCS) [63]
- Disability measured with the Oswestry Disability Index (ODI) [64]
- Depressed mood assessed by the Hospital Anxiety and Depression Scale (HADS) [65]

#### Treatment fidelity

To assess treatment fidelity and treatment protocol adherence, we will use a triangulation procedure that combines two different approaches based on Toomey et al. [66], and a third additional strategy to assess dosage. Firstly, the Study PT will use a study-specific manual as support for each core session when delivering the intervention and note on a physiotherapist self-report checklist which treatment components have been used in each session. Secondly, to ensure that the main components of the intervention described in the manual are included, audio-recordings will be made of core sessions and a random selection of these recordings will be evaluated by an assessor experienced in person-centredness and cognitive behavioural therapy (CBT). Thirdly, the treatment dose will be assessed by the number of sessions attended and length of each session in minutes reported by the Study PT, to address an additional aspect of fidelity and adherence [67].

#### Process and resource feasibility

Process feasibility [68] will be administered by the following variables: recruitment, intervention use, and acceptability. Recruitment aspects include the percentage of patients eligible after the screening procedure, as well as the reasons for declining participation or dropping out of the study. Patients who withdraw from the study will also be asked if data collected up until the time point of the withdrawal can be used in the study. Intervention use will be calculated as the ratio of the number of completed treatment sessions versus the number of planned sessions. Acceptability with the digital format and intervention will be evaluated using a semistructured interview at the end of the intervention where all intervention participants will be asked about their experience. The Study PT will answer a questionnaire with specific questions about their experience with the intervention. Furthermore, all study participants and assessors will be asked to complete study-specific questionnaires about their thoughts on the digital data collection format (including both PROMs and physical capacity tests) and using the accelerometer. The self-reported physical activity level at screening will be compared with accelerometer data at baseline to evaluate the chosen questionnaire’s ability to detect patients with a low level of physical activity. Treatment safety will be addressed by the type and frequency of possible adverse events during the intervention collected by the Study PT after each session.

### Sample size

Based on previous literature, the minimum sample size for assessing process and resource feasibility is *n* = 24 [68, 69]. The justifications for this sample size are based on a rationale for the feasibility, precision in the mean and variance, and regulatory considerations. We will include 15 patients in each group (*n* = 30 in total) to leave some margin for withdrawals.

### Statistical methods

Feasibility data will be analysed and reported descriptively. Demographic and clinical data will most likely be reported using a nonparametric approach, such as proportions and medians together with adequate approaches for describing variability, such as interquartile ranges or 95% confidence intervals. Similarly, we will also use nonparametric inferential tests for tentative analyses of efficacy and changes in outcomes within and between groups. In addition, data from the repeated measures will be plotted and analysed visually regarding changes in slope, medians, variability and regarding systematic patterns in these factors within and across participants [70]. The significance level will be set at a *p* value of 0.05. Numeric data will be analysed using the latest versions of IBM SPSS Statistics and/or RStudio. Accelerometer data will be analysed using ActiLife version 6 software. A wear time of a minimum of 10 h per day for at least 4 days will be considered valid [71]. Physical activity data will be reported as average steps per day, as well as minutes per week in each intensity category. We will calculate the proportions of patients who reached < 5000 steps per day (sedentary lifestyle), 5000–7499 steps per day (low active), 7500–9999 (somewhat active), and ≥ 10,000–12,499 steps per day (active) [72]. The proportion of participants in each physical activity level category as well as the proportion reaching the recommendations for health-promoting physical activity made by the WHO will be presented numerically and graphically. Data from the individual telephone interviews with the intervention participants will be transcribed verbatim. The text material will be analysed using inductive content analysis with a descriptive manifest approach.

## Discussion

Persons with physical activity levels below global recommendations have a substantially increased risk of developing poor overall health in comparison to those who are sufficiently active [38]. Therefore, increasing health behaviours such as physical activity is a public health priority. Patients undergoing lumbar spine surgery are less likely to meet recommendations for healthy physical activity at long-term follow-up after surgery compared to population norms [4]. Moreover, higher anxiety and fear-avoidance beliefs about pain are associated with lower physical activity (measured as step counts) in LSS patients specifically [16]. To our knowledge, this study will be the first of its kind to evaluate the feasibility of a person-centred and digital health-promoting intervention aiming to increase physical activity for risk patients undergoing decompression surgery for LSS.

In addition, Get Back aims to move away from a one size fits all approach to a person-centred approach in terms of both treatment and treatment outcomes. Given the multidimensional complexity of physical functioning in LSS, Get Back_feasibility_ will use a combination of PROMs, physical capacity tests, and accelerometer data to capture a comprehensive picture of patient functioning following surgery.

Based on the results of this feasibility study, the intervention will be refined by revisiting both the content and study procedure prior to the start of a randomized controlled trial evaluating the effect of the intervention. In the long term, if the intervention shows similar or improved outcome effects compared to those in the control group (receiving usual physical therapy), this could bring important knowledge to the field of lumbar spine rehabilitation. Furthermore, the intervention could facilitate equal rehabilitation by supporting the use of digital solutions that can benefit patients with long travel distances to rehabilitation centres.

## Trial status

Start of study enrolment April 17th, 2023.

### Supplementary Information

**Additional file 1.** SPIRIT checklist.

**Additional file 2.** Data management information (a complement to the SPIRIT checklist)

## Data Availability

Not applicable, as this is a study protocol which does not contain any data. Additional data management information: Please see Additional file 2.

## Abbreviations

LSS: Lumbar spinal stenosis
CBT: Cognitive behavioural therapy
PROM: Patient-reported outcome measure
WHO: World Health Organization
TSK: Tampa Scale of Kinesiophobia
PCS: Pain Catastrophizing Scale
PT: Physical therapist
BCT: Behaviour Change Technique
MVPA: Moderate to vigorous physical activity
