# Supplementary material for: Get Back, a person-centred digital programme targeting physical activity for patients undergoing spinal stenosis surgery—a study protocol of a randomized feasibility study
Source: Pilot Feasibility Stud. 2024 Jan 26;10:16. doi: 10.1186/s40814-023-01433-9 (PMC10811854; doi:10.1186/s40814-023-01433-9)
Supplement: Supplementary file 2 — Additional file 2. Data management information (a complement to the SPIRIT checklist) [file 40814_2023_1433_MOESM2_ESM.pdf]

Additional file 2.

Data management information (a complement to the SPIRIT checklist)

Item 19: Patients will be recruited at two spine clinics in Stockholm respectively Gothenburg, according to the procedure described in the protocol. Each research participant is given a unique code number from the recruiter at site when included. The study participants' social security numbers and unique code numbers are registered in a file (a code key) at the recruiting unit during ongoing data collection. The code key will be kept in a locked file or locked space on site during this time. On documents included in the data collection, only the code number is visible. The collected data is mainly registered digitally in an application (BASS, provided by Karolinska Institutet), thus entered in the application directly on site by the independent observer during baseline and follow-up assessments and by patients during the weekly monitoring. Patient data collected via the BASS application is stored on a safe server at Karolinska Institutet during the data collection. A data processing agreement has been established between the research principals and BASS, which gives BASS permission to process personal data on behalf of the research principals. Data collected through BASS is protected in accordance with legislation and regulations for sensitive personal data (including two-factor authentication and access control). At the end of the study, the Data Processor (BASS, Karolinska Institutet) hands over the data to the entity responsible for research (Sophiahemmet University) and deletes it from their server, according to the entity's wishes. Research data in paper format such as screening lists is converted to a digital format and entered in a data file stored on Sunet Drive (for detailed information on Sunet Drive please see below). During ongoing data collection paper data is stored in a locked cabinet at each study site. Recorded data will be transcribed without mentioning participants by name. As soon as this is done, the audio recordings will be deleted from the recording device after transfer to Sunet Drive.

Sophiahemmet University use the Sunet Drive storage solution for research data, which is a cloud solution offered via Sunet. The storage solution meets basic legal requirements and IT security. It is structured and configured to ensure technical compliance in terms of information security and the EU's General Data Protection Regulation (GDPR). It is built to protect research data against other actors or nations' possible decisions that could jeopardize its integrity by being based within Sweden's borders. The supplier has a statutory duty of confidentiality regarding the data stored. Sunet Drive complies with the Swedish Civil Contingencies Agency's guidelines for security measures in information systems for government agencies. All data is stored in Sunet's data center in Sweden, which meet the highest standards of secure operating space. Sunet Drive uses Sunet Storage Object Storage (StAAS) via an S3 interface.

Only researchers from the research group will have access to data and transcribed material. Administrative documents, code lists, data, and transcribed material will be stored in accordance with the Archives Act (1990:782) in locked files and cabinets at Sophiahemmet University for at least 10 years after the data collection is completed, which will be reported to the data protection officer at Sophiahemmet University. Two researchers from the research group will thoroughly go through the final dataset and do range checks, look for typos etc. A data management plan has been established and will be continuously updated during the study process. This information is also available in the approved ethical application of the study.

Item 25: In case of important protocol modifications during the feasibility study, the modification will first be processed in the steering group and with co-researchers before any decisions will be taken. Depending on the extent of the modification an update of the data management plan and on clinicaltrials.gov will be conducted. A new trial registration and a new study protocol for the larger

randomized controlled trial including modification based on the results from the feasibility phase will be formulated and submitted to a scientific journal.

Item 27 and 29: Potential and enrolled participants will be registered on screening lists by the recruiter at each recruiting site. Age, gender and self-reported physical activity level from eligible participants who declined participation in the study will be collected on the screening list to later enable interpretation regarding external validity of our findings. This have been stated and approved in the ethical application of the study (Dnr: 2022-07104-01). The screening lists will be handled in accordance with the EU's General Data Protection Regulation (GDPR). Safety procedure regarding data during collection at site is described under item 19 above.

All our research data (survey responses, recorded interviews, test results from functional capacity tests and data from motion sensors) are identified as sensitive research data. Personal data will be handled in accordance with GDPR. Only researchers from the research group will have access to data and transcribed material after the data collection is finalized. The data in the study will be pseudonymised (coded) personal data, and Swedish legislation prohibits us from sharing this completely open. Data is available upon request, which will be handled according to the relevant legislation. In most cases, this will require a data processing agreement or similar with the recipient of the data. All data processing is conducted in coded form and the data is presented at a group level so individual participants cannot be identified. Data storage for maintenance of confidentiality after the trial is described more under item 19 above.

Item 31a: Results of the study will be published open access in international peer review scientific journals, presented at international conferences, and presented at a national level through the Swedish Society of Spinal Surgeons, the Swedish Chapter of IASP, the Swedish Physiotherapist Association and through the broad network covered by the authors.
